# *Lepidium meyenii* Walp (Maca)‐derived extracellular vesicles ameliorate depression by promoting 5‐HT synthesis via the modulation of gut–brain axis

**DOI:** 10.1002/imt2.116

**Published:** 2023-06-04

**Authors:** Rui Hong, Lan Luo, Liang Wang, Zhao‐Li Hu, Qi‐Rong Yin, Ming Li, Bin Gu, Bin Wang, Tao Zhuang, Xin‐Yue Zhang, Yuan Zhou, Wan Wang, Lin‐Yan Huang, Bing Gu, Su‐Hua Qi

**Affiliations:** ^1^ School of Medical Technology Xuzhou Medical University Xuzhou China; ^2^ School of Pharmacy Xuzhou Medical University Xuzhou China; ^3^ Laboratory Medicine, Guangdong Provincial People's Hospital Guangzhou China; ^4^ School of Medical Informatics and Engineering Xuzhou Medical University Xuzhou China; ^5^ Centre for Precision Health, School of Medical and Health Sciences Edith Cowan University Perth Western Australia Australia; ^6^ Research Center for Biochemistry and Molecular Biology and Jiangsu Key Laboratory of Brain Disease Bioinformation Xuzhou Medical University Xuzhou China; ^7^ Department of Laboratory Medicine Affiliated Hospital of Xuzhou Medical University Xuzhou China

**Keywords:** 5‐HT, brain‐derived neurotrophic factor, depression, gut–brain axis, *Lepidium meyenii* Walp‐derived extracellular vesicles, unpredictable chronic mild stress

## Abstract

Depression is a common and debilitating condition for which effective treatments are needed. *Lepidium meyenii* Walp (Maca) is a plant with potential medicinal effects in treating depression. Recently, there has been growing interest in plant‐derived extracellular vesicles (EVs) due to their low toxicity and ability to transport to human cells. Targeting the gut–brain axis, a novel strategy for depression management, may be achieved through the use of Maca‐derived EVs (Maca‐EVs). In this study, we successfully isolated Maca‐EVs using gradient ultracentrifugation and characterized their shape, size, and markers (CD63 and TSG101). The *in vivo* imaging showed that the Dil‐labeled Maca‐EVs crossed the brain–blood barrier and accumulated in the brain. The behavioral tests revealed that Maca‐EVs dramatically recovered the depression‐like behaviors of unpredictable chronic mild stress (UCMS) mice. UCMS mice fecal were characterized by an elevated abundance of *g_Enterococcus*, *g_Lactobacillus*, and *g_Escherichia_Shigella*, which were significantly restored by administration of Maca‐EVs. The effects of Maca‐EVs on the altered microbial and fecal metabolites in UCMS mice were mapped to biotin, pyrimidine, and amino acid (tyrosine, alanine, aspartate, and glutamate) metabolisms, which were closely associated with the serotonin (5‐HT) production. Maca‐EVs were able to increase serum monoamine neurotransmitter levels in UCMS mice, with 5‐HT showing the most significant changes. We further demonstrated that 5‐HT improved the expression of brain‐derived neurotrophic factor, a key regulator of neuronal plasticity, and its subsequent activation of TrkB/p‐AKT signaling by regulating the GTP‐Cdc42/ERK pathway. These findings suggest that Maca‐EVs enhance 5‐HT release, possibly by modulating the gut–brain axis, to improve depression behavior. Our study sheds light on a novel approach to depression treatment using plant‐derived EVs.

## Highlights

- We successfully isolated Maca‐EVs using ultracentrifugation and tested its antidepressant effects in the unpredictable chronic mild stress mice.
- Maca‐EVs increased serum serotonin (5‐HT) levels probably via the gut microbiota and metabolism modulation.
- The antidepressant activity of Maca‐EVs may be attributed to the enhanced serum 5‐HT levels and subsequent activation of the GTP‐Cdc42/ERK and BDNF/TrkB/AKT signaling pathway.

## Graphical abstract

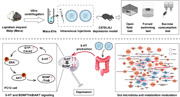

## INTRODUCTION

Depression is a mental disorder that affects 280 million individuals globally, including 5.0% of adults and 5.7% of the elderly. Severe depression can lead to suicide, which is the fourth leading cause of death in populations between the ages of 15–29. Recent data released by the WHO have demonstrated that a 25% increase in the global prevalence of depression due to the spread of COVID‐19 [1]. It has been found that the dysfunction in excitatory synapses, microglia, neurotransmitters, neuroimmune, and neuroinflammatory actions may contribute to the development of depression [2, 3, 4]. Consequently, drugs that regulate neurotransmitters such as serotonin (5‐HT), norepinephrine (NE), dopamine (DA), and others are included in the clinical management of depression [5]. Traditional antidepressants such as monoamine oxidase inhibitors, tricyclic, selective 5‐HT reuptake inhibitor, 5‐HT, and noradrenaline reuptake inhibitors have been shown to have particular antidepressant efficacy. However, their adverse effects such as sexual dysfunction, nausea/vomiting, weight changes, sleep disruption, and easy addiction could not be ignored [6]. Furthermore, almost 50% of patients with depression do not respond to antidepressant treatment [7]. Thus, there is an unmet need for the development of safe and effective antidepressant.

Over the past decade, a growing body of evidence has highlighted that the gut–brain axis action is essential in the pathophysiological development of depression [8]. This axis refers to the bidirectional interactions between the gut and brain, facilitated by neural (enteric, sympathetic, and vagus nerves), immune (inflammatory cytokines and cells), and chemical (microbiota metabolites and neurotransmitters) signals. In individuals with depression, the activity of the enteric and sympathetic nervous systems is elevated. Recent findings have shown that the depression‐like behaviors induced by lipopolysaccharide or “depression‐related” microbes injection in mice could be alleviated by subdiaphragmatic vagotomy, demonstrating the potential role of the vagus nerve in depression [9, 10]. Additionally, depression is characterized by elevated levels of peripheral proinflammatory cytokines or chemokines, such as tumor necrosis factor (TNF‐α), interleukin‐6 (IL‐6), and IL‐1β, and activation of immune cells such as neuron cells in the brain, dendritic cells, and innate lymphoid cells in the gut, monocytes, and macrophages in the systemic circulation [11]. Furthermore, certain gut microbiota can produce specific metabolites (e.g., tryptophan metabolites, trimethylamine‐*N*‐oxide, and short‐chain fatty acids), neuroactive modulators (e.g., 5‐HT, aminobutyric acid, brain‐derived neurotrophic factor [BDNF], and glia‐derived neurotrophic factor), and other factors that are secreted by intestinal cells or from bacteria decomposition [12]. Therefore, the gut–brain axis is emerging as a novel target for drug development to treat depression.

*Lepidium meyenii* Walp, also known as Maca, has been cultivated in the Andean region for at least 2000 years and successfully introduced in China since 2002, including Yunnan, Xinjiang, Jilin, and Tibet Province [13]. Maca is a traditional edible medicine plant renowned for its benefits in hormone balance, regulation of sexual dysfunction, and energizing effects [13]. Maca is rich in essential nutrients and bioactive components that vary depending on its color and type, resulting in diverse biological functions [14]. The three most used and studied types are yellow, red, and black Maca. Recently, Maca and its extract have garnered significant attention due to its potential neuroprotective and antidepressant effects. Studies have shown that Maca macamide repairs the corticosterone‐induced hippocampal impairments through its anti‐inflammatory, neurotrophic, and synaptic protection features [15]. Additionally, yellow, red, and black Maca have demonstrated antidepressant activity in ovariectomized mice [16]. Furthermore, the petroleum ether extract of Maca exhibited antidepressant action by activating the noradrenergic and dopaminergic systems and inhibiting oxidative stress in the mouse brain [17]. Nevertheless, the limited ability of Maca and its extract to cross the blood–brain barrier (BBB) remarkably hinders their potential as therapeutic agents. Plant‐derived extracellular vesicles (PDEVs), similar to animal‐derived extracellular vesicles (EVs), have been shown to participate in human cell‐to‐cell communication, exerting various biological functions, such as innate immunity and inflammation modulation [18]. The small size of EVs (<200 nm) enables them to cross the BBB freely, making them promise new candidates for translational applications [19].

In this study, we successfully isolated and purified EVs from Maca (Maca‐EVs) based on the minimal criterion of EVs characterizations. Behavioral experiments indicated the Maca‐EVs had remarkable antidepressant effects in the unpredictable chronic mild stress (UCMS) mice. The antidepressant activity of Maca‐EVs may be attributed to the enhancement of serum 5‐HT levels and subsequent activation of the BDNF/TrkB/AKT axis. Possible mechanisms for the increased serum 5‐HT levels include modulation of the gut–brain axis, among others.

## RESULTS

### Isolation and characterization of Maca‐EVs

**Figure 1:**
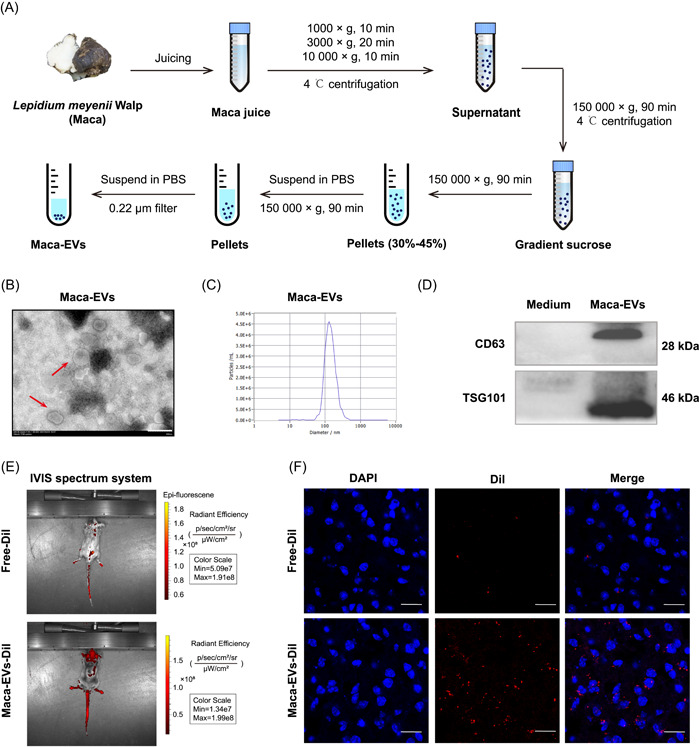
Isolation and characterization of Maca‐EVs. (A) Flowchart on the isolation of EVs from *Lepidium meyenii* Walp (Maca). (B) Transmission electron microscope image on the structure of Maca‐EVs (red arrow). Scale bar: 200 nm. (C) NTA analysis revealed that the size of Maca‐EVs was around 134 nm. (D) Western blot analysis on the expressions of EVs markers (CD63 and TSG101) in the Maca medium and Maca‐EVs. (E) Representative images on the *in vivo* location of Free‐Dil or Maca‐EVs‐Dil in the brain of mice by the IVIS optical imaging system. (F) Representative immunofluorescence images on the brain tissue of mice that killed 12 h later after the injection of Free‐Dil or Maca‐EVs‐Dil. Scale bar: 100 μm. DAPI, 4′,6‐diamidino‐2‐phenylindole; EV, extracellular vesicle; IVIS, *in vivo* imaging system; NTA, nanoparticle tracking analysis; PBS, phosphate‐buffered saline.

Maca‐EVs were isolated from Maca juice using gradient ultracentrifugation as described in Figure 1A. Transmission electron microscopy imaging and nanoparticle tracking analysis revealed that Maca‐EVs were irregular‐shaped membrane‐enclosed vesicles (Figure 1B) with an average diameter of 134 nm (Figure 1C). Moreover, Maca‐EVs expressed higher EVs markers CD63 and TSG101 than the Maca medium (Figure 1D), indicating the successful isolation of EVs from Maca juice. Next, we investigated the *in vivo* uptake of Maca‐EVs in C57BL/6J mice using an IVIS Spectrum imaging system. Intravenously administrated Dil labeled Maca‐EVs (Maca‐EVs‐Dil) accumulated the mice brain with robust red fluorescence signals compared with the free‐Dil administrated mice (Figure 1E). These mice were killed 12 h later after Maca‐EVs‐Dil or free‐Dil administration, and brain‐frozen sections were prepared. Immunofluorescence staining confirmed that Maca‐EVs could pass through the BBB and accumulate in the brain (Figure 1F).

### Maca‐EVs reverse the depressive‐like behaviors in the UCMS mice

**Figure 2:**
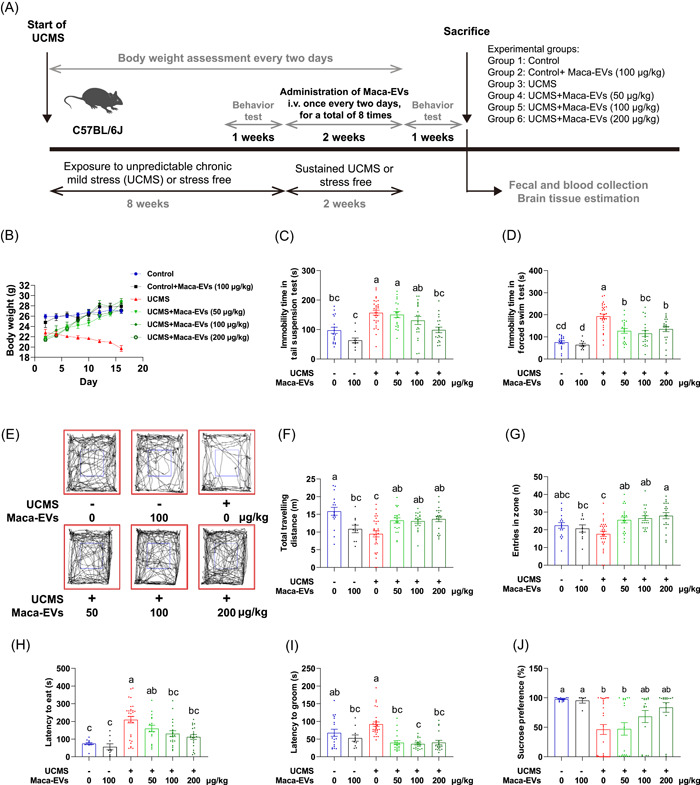
Antidepressive effects of Maca‐EVs in the UCMS mice. (A) Schematic illustration on the UCMS model establishment and drug administration in the present study. (B) Body weight changes of control and UCMS mice with indicated Maca‐EVs treatments at different time points. Effect of Maca‐EVs on the immobility time in tail suspension test (C) and in forced swimming test (D) of control and UCMS mice with indicated Maca‐EVs treatments. (E) Representative open field test photos of control and UCMS mice with indicated Maca‐EVs treatments. Quantification on the total traveling distance (F) and entries in zone (G) of the open field test. Effect of Maca‐EVs on the latency to eat (H) and to groom (I), and percentage of sucrose preference (J) of control and UCMS mice with indicated Maca‐EVs treatments. All data are presented as mean ± SEM (*n* = 15–30 experiments for each group). Significance was evaluated by RM two‐way analysis of variance (ANOVA) followed by Tukey's multiple comparisons tests in (B), and significance was evaluated by ordinary one‐way ANOVA followed by Tukey's multiple comparisons test in (C, F, G), Brown–Forsythe and Welch ANOVA tests followed by the Tamhane T2 multiple comparisons test in (D), and the Kruskal–Wallis test followed by Dunn's multiple comparisons test in (H, I, J). Means denoted by a different letter indicated significant differences between groups (*p* < 0.05). EV, extracellular vesicle; RM, repeated measure; SEM, scanning electron microscope; UCMS, unpredictable chronic mild stress.

Next, we assessed the antidepressant‐like effect of Maca‐EVs *in vivo*. We applied daily UCMS in C57BL/6J mice for 8 weeks to establish the depression model (Figure 2A). Stress‐free treated mice were included as controls. UCMS dramatically deceased the body weight (Figure S1A), increased immobility time in the tail suspension test (Figure S1B) and forced swim test (Figure S1C), increased latency to groom and eat (Figure S1D,E), and decreased sucrose consumption (Figure S1F) in the mice. There were no significant changes in the time of open arm and close arm, indicating a depression model, but not an anxiety model (Figure S1G,H). Control mice were randomly divided into two groups: control and control injected with Maca‐EVs (100 μg/kg). UCMS mice were randomly divided into four groups: UCMS and UCMS injected with different doses of Maca‐EVs (50, 100, and 200 μg/kg). Maca‐EVs were administrated once every 2 days for a total of eight times in 2 weeks. Notably, we continuously applied daily UCMS to the mice during the Maca‐EVs treatment. Fecal and blood samples were collected from the mice, and the mice were killed after the behavioral tests were completed (Figure 2A). Body weights of the mice were continually declined in UCMS mice, but were comparable to the control mice administrated with Maca‐EVs (200 μg/kg) (Figure 2B). Maca‐EVs administration improved depressive‐like behaviors in UCMS mice, as shown by the decline in the extended immobility time in the tail suspension test (Figure 2C) and the forced swim test (Figure 2D). The open field test (Figure 2E) showed that Maca‐EVs administration improved exploratory behavior with elevated traveling distances (Figure 2F) and entry frequency in the zone (Figure 2G) of UCMS mice. The sucrose consumption test demonstrated a significant reduction in the latency to eat (Figure 2H) or groom (Figure 2I) and an increase in sucrose preference (Figure 2J) in Maca‐EVs ‐administrated UCMS mice compared with the nontreated UCMS mice. These data suggested the therapeutic potential of Maca‐EVs in treating depression.

### Changes in the fecal microbiota in Maca‐EVs treated or nontreated control and UCMS mice

**Figure 3:**
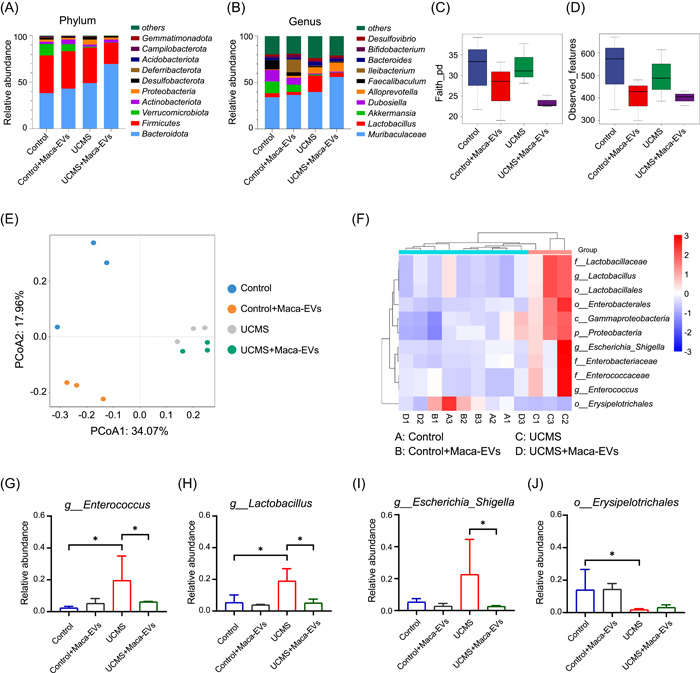
Effects of Maca‐EVs on the microbiota composition in Control and UCMS mice feces. Mice were killed when the behavior tests were accomplished, and feces were collected for 16S rRNA sequencing analysis. Control, Control + Maca‐EVs, UCMS, and UCMS + Maca‐EVs groups were included in the present study (*n* = 3 experiments for each group). Relative abundance of microbiota (top 10) at phylum (A) and genus (B) levels in the different groups. Alpha diversity regarding the index of Faith's phylogenetic diversity metric (pd) (C) and observed features (D) in the different groups. (E) Principal coordinate analysis (PCoA) based on the Bray‐curtis distance matrixes between sample groups. (F) Linear discriminant analysis effect size (LEfSe) was performed to compare microbial composition at 16S rDNA operational taxonomic unit (OTU) level between sample groups. A total of 11 OTUs that significantly differed between New_Control and UCMS groups (*p* < 0.05; linear discriminant analysis [LDA] > 4.0) were shown. (G–J) The abundance changes of typical bacteria that were significantly differed between New_Control and UCMS among sample groups. EV, extracellular vesicle; rDNA, recombinant DNA; rRNA, ribosomal RNA; UCMS, unpredictable chronic mild stress.

We next investigated the dynamic changes of gut microbiota in mice from across four groups (Control, Control + Maca‐EVs, UCMS, and UCMS + Maca‐EVs), using 12 fecal samples. A total of 718,971 high‐quality 16S ribosomal RNA (rRNA) sequences were obtained with an average of 59,914 reads per sample. After the rarefaction of sample to an equal sequencing depth (56,402 reads per sample) and clustering, 676,824 sequences were grouped into 1434 operational taxonomic units (OTUs) for the downstream analysis. At the phylum level, Bacteroidota and Firmicutes (Figure 3A) were the most abundant in all groups, while at the genus level, *Muribaculaceae* was the most abundant in all groups (Figure 3B). The genus *Lactobacillus* varied among groups, with a significant decrease in Control, Control + Maca‐EVs, and UCMS + Maca‐EVs groups compared with UCMS group (*p* < 0.05, Figure 3B). In contrast, the abundance of *Akkermansia*, *Dubosiella*, and *Faecalibaculum* was significantly lower in the UCMS group than that in the other groups (Figure 3B). These data indicated that the genera *Lactobacillus*, *Akkermansia*, *Dubosiella*, and *Faecalibaculum* might be associated with the effect of UCMS and Maca‐EVs.

We also investigated the effect of Maca‐EVs on microbial diversity and found that microbial richness, as indicated by the Faith‐pd and Observed features index, was higher in the Maca‐EVs groups (Control + Maca‐EVs and UCMS + Maca‐EVs), compared with Control and UCMS groups (Figure 3C,D). Only the Faith‐pd index in Control + Maca‐EVs group was significantly lower than that in the Control group (*p* < 0.05, Figure 3D). The community richness of the UCMS group relative to the Control group decreased but not significantly. To evaluate the changes of β‐diversity of gut microbiota across different groups, we conducted a principal coordinate analysis based on the Bray‐curtis distance matrixes (Figure 3E) and partial least squares‐discriminant analysis (PLS‐DA) (Figure S2B). We found that the four groups were clustered into distinct groups with β‐diversity estimates, as assessed by the ANOSIM tests (*R* = 0.772, *p* = 0.001).

To obtain a deeper insight of microbiota alterations upon Maca‐EVs administration, we performed linear discriminant analysis effect size (LEfSe) to compare microbial composition at 16S recombinant DNA OTU level. The Venn diagram (Figure S2A) showed that 444 OTUs were shared between Control and Control + Maca‐EVs groups, while 494 were unique to Control group and 222 to Control + Maca‐EVs group. Between Control and UCMS groups, 578 OTUs were shared, while 360 were unique to Control group and 375 to UCMS group. Between UCMS and UCMS + Maca‐EVs groups, 471 OTUs were shared, while 382 were unique to UCMS group and 209 to UCMS + Maca‐EVs group. We grouped Control, Control + Maca‐EVs, and UCMS + Maca‐EVs into a new group called New_Control. A total of 11 OTUs that significantly differed between New_Control and UCMS groups (*p* < 0.05; linear discriminant analysis >4.0) were shown in Figure 3F, of which 1 OTU was enriched in New_Control group and 10 in the UCMS group. Among those differential OTUs, 2 OTUs belong to order Lactobacillales, which are Lactobacillaceae and Enterococcaceae at the family level. To further investigate which OTUs were associated with depressive‐like symptoms, we selected four typical bacteria (Figure 3G–J) that were significantly differed between New_Control and UCMS groups to see their abundance changes among the four groups. Furthermore, we selected four typical bacteria that were significantly different between the Control and UCMS groups (Figure S2C–F).

### Changes in the fecal metabolic signatures of Maca‐EVs treated or nontreated control and UCMS mice

**Figure 4:**
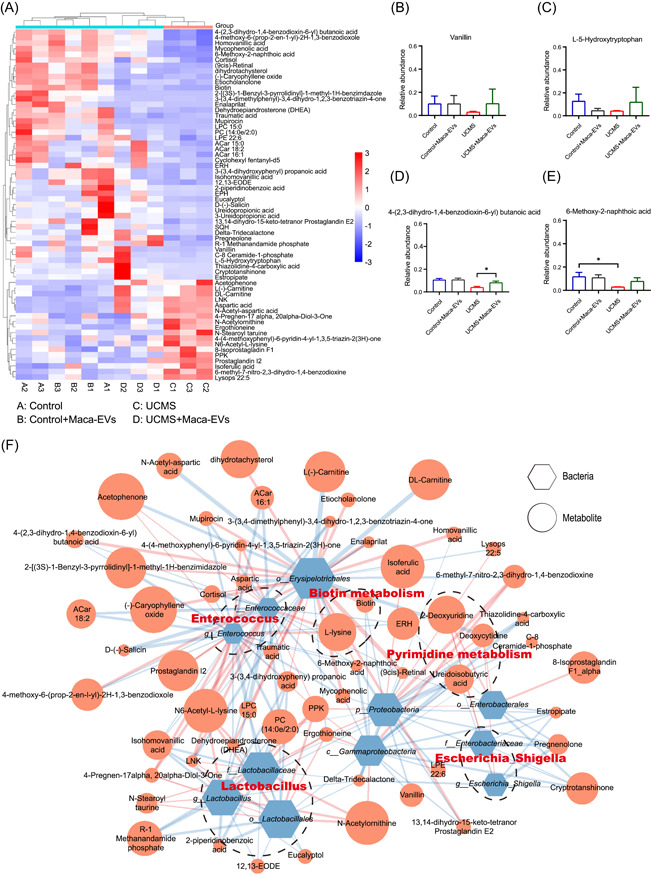
Effects of Maca‐EVs on the metabolomics in control and UCMS mice feces. (A) Differential metabolites between New_Control and UCMS group (VIP > 1, *p* < 0.05, |log FC| > 1) in pos mode. (B–E) The levels of four typical metabolites that were significantly differed between New_Control and UCMS groups among sample groups. (F) Reciprocal interactions between altered gut bacteria and serum metabolites identified by a co‐occurrence network based on Spearman correlation analysis in pos mode. EV, extracellular vesicle; UCMS, unpredictable chronic mild stress. VIP, variable importance in projection.

Given gut microbiome often modulates the host metabolic pathway, here we used gas chromatography–mass spectrometry (GC‐MS)‐based metabolomics to compare the metabolic characteristics of the four groups. To detect more metabolites, nontargeted metabolomics was performed in two modes, positive ion (pos) and negative ion (neg). Principal components analysis and PLS‐DA were conducted to evaluate the changes of fecal metabolic signatures across different groups (Figure S3A–D). Specifically, Figure S3A,S3B was obtained from pos mode, Figure S3C,S3D was obtained from neg mode. Overall, the results showed spatial separation of metabolic signatures among the four groups, particularly between New_Control group and UCMS group, indicating compositional differences between them. In pos mode, 63 differential metabolites were identified between New_Control and UCMS group (variable importance in projection [VIP] > 1, *p* < 0.05, |log FC| > 1) (Figure 4A). These included Vanillin, l‐5‐hydroxytryptophan, 4‐(2,3‐dihydro‐1,4‐benzodioxin‐6‐yl) butanoic acid, 6‐methoxy‐2‐naphthoic acid enriched in New_Control group, and Prostaglandin I2, Lysops 22:5, *N*‐acetylornithine, *N*‐acetyl‐aspartic acid enriched in UCMS group. In neg mode (Figure S3G), 47 differential metabolites were found between New_Control and UCMS group (VIP > 1, *p* < 0.05, |log FC| > 1). Metabolites enriched in New_Control group included *N*‐isobutyrylglycine, *N*‐acetylalanine, pantothenic acid, 2‐keto‐4‐methylthiobutyric acid, and so forth, while metabolites enriched in the UCMS group included lysophosphatidylethanolamine 18:1, Apigenin, Saccharin, 5‐methoxysalicylic acid, and so forth. We selected four typical metabolites (Vanillin, l‐5‐hydroxytryptophan [5‐HTP], 4‐(2,3‐dihydro‐1,4‐benzodioxin‐6‐yl) butanoic acid, and 6‐methoxy‐2‐naphthoic acid) that were significantly differed between the New_Control and UCMS groups and analyzed their changes among the four groups (Figure 4B–E). Pathway enrichment analysis indicated that UCMS caused major alterations in metabolic pathway related to biotin, pyrimidine, tyrosine, alanine, aspartate, and glutamate metabolism (Figure S3E). By tryptophan hydroxylase converts tryptophan into 5‐hydroxytryptophan (5‐HTP), which is then further converted to 5‐HT by 5‐HTP decarboxylase [20] (Figure S3F). In our metabolomics analysis, we found that the amount of 5‐HTP in this pathway was significantly lower in the UCMS group but higher in the UCMS + Maca‐EVs group (Figure 4C), suggesting that UCMS may affect 5‐HT biosynthesis. Taken together, these results suggest that metabolites are associated with distinct groups.

### Co‐occurrence network analysis between the gut microbiota and fecal metabolites

To investigate the potential reciprocal interactions between altered gut bacteria and metabolites, a co‐occurrence network was constructed based on Spearman correlation analysis (Figure 4F). In pos mode, we found that the genus *Lactobacillus* formed a strong co‐occurring relationship with fecal metabolites assigned to 2‐piperidinobenzoic acid, R‐1 methanandamide phosphate, polyphosphate kinase (PPK), isohomovanillic acid, and *N*6‐acetyl‐l‐lysine. The genus *Enterococcus* formed a strong co‐occurring relationship with fecal metabolites assigned to lysophosphatidylcholine (LPC) 15:0, D‐(‐)‐Salicin, ACar 16:1, 4‐Pregnen‐17,20‐Diol‐3‐One, leucine (L)‐asparagine (N)‐lysine (K) (LNK), *N*6‐acetyl‐l‐lysine. The genus *Escherichia_Shigella* formed a strong co‐occurring relationship with fecal metabolites assigned to pregnenolone, delta‐tridecalactone, estropipate, 2‐deoxyuridine, and deoxycytidine. The genera *Lactobacillus* was positively correlated with PPK, *N*6‐acetyl‐l‐lysine, negatively correlated with 2‐piperidinobenzoic acid, R‐1 methanandamide phosphate, and isohomovanillic acid. The genus *Enterococcus* was positively correlated with 4‐Pregnen‐17,20‐Diol‐3‐One, LNK, and *N*6‐acetyl‐l‐lysine, negatively correlated with LPC 15:0, D‐(‐)‐Salicin and ACar 16:1. The genus *Escherichia_Shigella* was positively correlated with 2‐deoxyuridine and deoxycytidine, negatively correlated with pregnenolone, delta‐tridecalactone, and estropipate. Taken together, the altered *Enterococcus*, *Lactobacillus*, and *Escherichia_Shigella* were mapped to biotin and pyrimidine metabolism in pos mode. We can also see co‐occurrence network in neg mode which showed that the altered *Enterococcus*, *Lactobacillus*, and *Escherichia_Shigella* were mapped to tyrosine, alanine, aspartate, and glutamate metabolism (Figure S3F,G). Notably, these findings indicate that altered gut microbiota and metabolites formed a synergistic and node‐related co‐occurrence network between the New_Control and UCMS groups.

### Maca‐EVs enhance serum 5‐HT expressions in the UCMS mice

**Figure 5:**
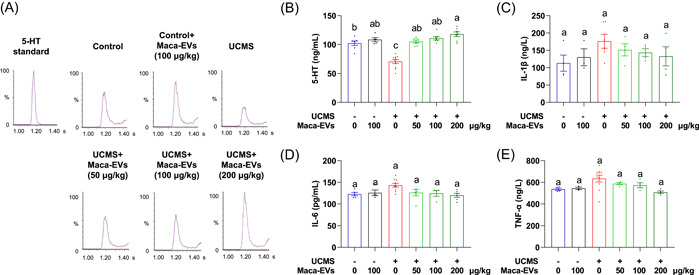
Serum monoamine neurotransmitters and inflammatory cytokines expressions analyses. (A) The UPLC‐MS/MS chromatograms of monoamine factors, including 5‐HT in the standard and serum of control and UCMS mice with indicated Maca‐EVs treatments. (B) Quantitative data on the concentrations of 5‐HT in the serum of control and UCMS mice with indicated Maca‐EVs treatments. Quantitative data on the concentrations of inflammatory cytokines, including IL‐1β (C), IL‐6 (D), and TNF‐α (E) in the serum of control and UCMS mice with indicated Maca‐EVs treatments. All data are presented as mean ± SEM (*n* = 3–11 experiments for each group). Significance was evaluated by ordinary one‐way analysis of variance (ANOVA) followed by Tukey's multiple comparisons test in (B, C, E) and the Kruskal–Wallis test followed by Dunn's multiple comparisons test in (D). Means denoted by a different letter indicated significant differences between groups (*p* < 0.05). 5‐HT, serotonin; EV, extracellular vesicle; IL, interleukin; MS, mass spectrometry; SEM, scanning electron microscope; UCMS, unpredictable chronic mild stress; UPLC, ultraperformance liquid chromatography.

As the identified altered metabolisms were closely associated with 5‐HT production, we next investigated whether Maca‐EVs modulate the monoamine neurotransmitters production. We collected the mice serum after completing the behavioral tests to measure the levels of the three major monoamine neurotransmitters (5‐HT, NE, and DA) via HPLC. The signals of NE and DA were too low to quantify. We observed significantly downregulated serum 5‐HT levels in the UCMS mice compared with the control mice, confirming the successful establishment of the UCMS model (Figure 5A,B). Maca‐EVs administration dramatically increased serum 5‐HT levels in the UCMS model at all doses. The significantly elevated serum NE and DA levels in the UCMS mice were observed when treated with Maca‐EVs that started from 100 to 200 μg/kg doses. While the serum inflammatory cytokines IL‐1β, IL‐6, and TNF‐α levels were increased in the UCMS mice compared with the control mice, the differences were not significant (Figure 5C–E). At a dose of 200 μg/kg, Maca‐EVs significantly reduced TNF‐α levels in serum, while the decrease in IL‐1β and IL‐6 expression was not statistically significant. Collectively, the serum monoamine neurotransmitters levels, particularly 5‐HT, may contribute to the antidepressant effects of Maca‐EVs.

### Maca‐EVs attenuate the decrease in the BDNF expression by activating the GTP‐Cdc42/ERK signaling pathway in the hippocampus and cortex of UCMS mice

**Figure 6:**
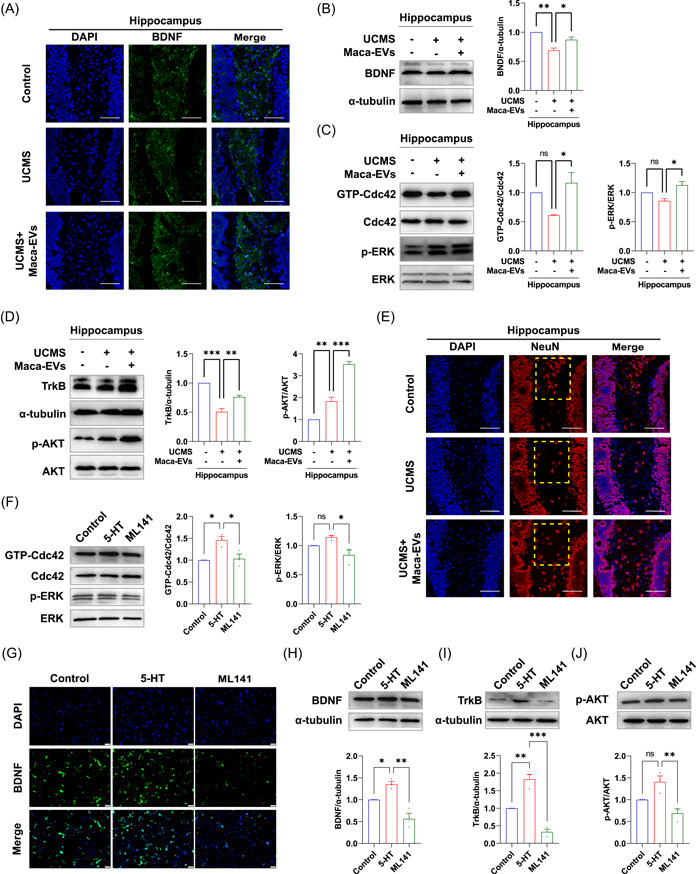
Maca‐EVs modulated the BDNF/GTP‐Cdc42/ERK and TrkB/AKT signaling in the hippocampus of mice via the 5‐HT. (A) Representative immunofluorescence images on the BDNF expressions in the hippocampus of control, Maca‐EVs treated (200 μg/kg), or untreated UCMS mice. Scale bar: 50 μm. Western blot images and quantitative data on the expressions of BDNF (B), Cdc42 enzyme activity, p‐ERK and ERK (C), TrkB, p‐AKT and AKT (D) in the hippocampus of control, Maca‐EVs untreated or treated (200 μg/kg) UCMS mice. (E) Representative immunofluorescence images on the NeuN cells in the hippocampus of control, Maca‐EVs treated (200 μg/kg), or untreated UCMS mice. The yellow dashed box indicated the dentate gyrus zone of hippocampus. Scale bar: 50 μm. (F) PC12 cells were treated with 5‐HT or ML141 (a GTP‐Cdc42 inhibitor) for 12 h and cultured for another 24 h. Western blot images and quantitative data on the expressions of GTP‐Cdc42, Cdc42, p‐ERK, and ERK in PC12 cells were shown. Immunofluorescence (G) and western blot analysis (H) and on the BDNF expressions in PC12 cells. Scale bar: 50 μm. Western blot images and quantitative data on the expressions of TrkB (I), p‐AKT, and AKT (J) in PC12 cells. All data are presented as mean ± SEM (*n* = 3 experiments for each group). Significance was evaluated by ordinary one‐way analysis of variance (ANOVA) followed by Tukey's multiple comparisons test in (B, C, D, F, H, I, J). ns, not significant, *p* > 0.05, *p < 0.05, **p < 0.01, and ***p < 0.001 between two indicated groups. 5‐HT, serotonin; BDNF, brain‐derived neurotrophic factor; DAPI, 4′,6‐diamidino‐2‐phenylindole; EV, extracellular vesicle; SEM, scanning electron microscope; UCMS, unpredictable chronic mild stress.

The coregulation of 5‐HT and BDNF signaling is well established, whereby 5‐HT activates the BDNF expression, which is essential for the growth and survival of 5‐HT neurons. Abnormal 5‐HT and BDNF signaling are associated with the progression of depression [21]. The administration of Maca‐EVs at a dose of 200 μg/kg significantly increased serum 5‐HT levels, promoting us to investigate whether it would enhance BDNF expression in the UCMS mice. Using the immunofluorescence staining and WB analysis, we confirmed the downregulation of BDNF expression in the hippocampus (Figure 6A,B) and cortex (Figure S4A,B) of the UCMS mice, which was attenuated by Maca‐EVs administration. To further explore the underlying mechanism, we examined the GTP‐Cdc42/ERK signaling pathway that regulates BDNF expression. The WB analysis revealed an upregulated ratio of GTP‐Cdc42/Cdc42 and p‐ERK/ERK in the hippocampus (Figure 6C) and cortex (Figure S4C) of the Maca‐EVs administrated UCMS mice compared with the saline‐administrated UCMS mice. These results suggest that Maca‐EVs attenuate the decrease in BDNF expression by triggering the GTP‐Cdc42/ERK signaling in the hippocampus and cortex of UCMS mice.

### Maca‐EVs mitigate the neuron number decline via modulating the TrkB/AKT signaling in the hippocampus and cortex of UCMS mice

Moreover, we explored whether the upregulated BDNF expression modulates neuron growth in UCMS mice. BDNF interacts with its receptor Tyrosine Kinase receptor B (TrkB) and initiates the downstream AKT signaling to maintain neuron growth. We observed downregulated TrkB expression and upregulated p‐AKT/AKT ratio in the UCMS mice, which were significantly rescued by Maca‐EVs administration (Figures 6D and S4D,E). The immunofluorescence staining revealed that the Maca‐EVs administration promoted neuron growth in the dentate gyrus zone of UCMS mice, which was declined (Figures 6E and S4F). Therefore, the data demonstrated that Maca‐EVs mitigated the declined number of neurons via modulating the TrkB/p‐AKT signaling in the cortex and hippocampus of UCMS mice.

### The 5‐HT induces BDNF expression and subsequent activation of TrkB/AKT signaling via modulating the GTP‐Cdc42/ERK pathway *in vitro*

To determine whether the 5‐HT activation regulates the activity of GTP‐Cdc42, we treated PC12 cells with ML141, a GTP‐Cdc42 inhibitor, and observed a significant decrease in the ratio of GTP‐Cdc42/Cdc42 and p‐ERK/ERK (Figure 6F). We also found that the upregulation of BDNF expression induced by 5‐HT was mitigated by the ML141 treatment (Figure 6G,H), indicating that 5‐HT stimulates BDNF expression through modulation of the GTP‐Cdc42/ERK pathway. Moreover, ML141 treatment blocked the subsequent TrkB/AKT signaling of 5‐HT, as evidenced by the declined TrkB expression and ratio of p‐AKT/AKT (Figure 6I,J). These data suggest that 5‐HT activates the GTP‐Cdc42/ERK pathway to stimulate BDNF expression and subsequent activation of TrkB/AKT signaling.

## DISCUSSION

To the best of our knowledge, we are the first to successfully isolate and characterize EVs from Maca. Maca‐EVs exhibited features similar to animal cell‐derived EVs and could pass through the BBB. Intravenous injection of Maca‐EVs noticeably reduced the depressive behaviors in UCMS mice. Our analysis, using 16S rRNA and GC‐MS‐based metabolomics, identified that Maca‐EVs altered the composition of depression‐related fecal microbiota and improved metabolisms associated with 5‐HT production in UCMS mice. This suggests the potential of Maca‐EVs to modulate the gut–brain axis. Furthermore, we found that Maca‐EVs significantly enhanced serum 5‐HT levels, which were decreased in UCMS mice, as determined by HPLC analysis. *In vivo* and *in vitro* experiments revealed that 5‐HT stimulates BDNF expression and consequent activation of BDNF/TrkB/AKT signaling, which may contribute to the antidepressant effects of Maca‐EVs.

Traditional Chinese Medicine (TCM) has long been widely used in the treatment of depression, particularly in Asia. The molecular mechanisms underlying the action of TCM involve the regulation of monoamine transmission, hypothalamic‐pituitary‐adrenal axis, neurotrophins, such as BDNF, as well as the number and function of synapses [22]. Maca extract has been reported to possess neuroprotective and antidepressant effects attributed to its antioxidant activity [23]. Later studies identified macamides as the active constituents responsible for the neuroprotection by inhibiting fatty acid amide hydrolase, which regulates neural progenitor cell proliferation [24]. In addition, Maca extract has been suggested to have anti‐inflammatory properties by reducing the inflammatory cytokine IL‐6 in Maca users [25]. However, the precise molecular mechanisms of Maca and its extract in treating depression remain to be elucidated.

Over the past decades, PDEVs, which are spherical‐, oval‐, or cup‐shaped vesicles containing biologically active elements, including lipids, proteins, nucleic acids, and secondary metabolites, have gained substantial attention in therapeutic drug development. Increasing *in vitro* and *in vivo* studies have shown that PDEVs possess anticancer, anti‐inflammatory, antioxidant, and regenerative activities [26]. Of note, Teng et al. identified that microRNA (miRNA) from ginger‐derived PDEVs can target the gut bacteria *Lactobacillus rhamnosus* genome to produce more indole‐3‐carboxaldehyde, which promotes IL‐22 secretion, enhancing gut barrier function [27]. Despite promising preclinical studies, the translation of PDEVs from bench to bedside is still in its infancy. At present, the University of Louisville has conducted three clinical trials (NCT04879810, NCT01668849, and NCT01294072) using four types of PDEVs (curcumin, ginger, aloe, and grape) [28]. However, the complete results of these clinical trials have yet to be reported. Therefore, significant efforts are needed to focus on the isolation, purification, physiochemical characterization, quality control, safety, and delivery routes of PDEVs [29].

Our group has previously isolated EVs from *Momordica charantia*, a fruit of the Cucurbitaceae plant, and demonstrated its therapeutic potential in treating cerebral ischemia/reperfusion [30] and radiation‐induced heart disease [31]. Hence, we aimed to isolate EVs from black Maca and investigate their antidepressant potency in a UCMS mice model, along with the relevant mechanisms. We successfully isolated Maca‐EVs by gradient centrifugation, which exhibited features similar to animal cell‐derived EVs (Figure 1A–D). We used the lipophilic fluorescent dye Dil, which is widely used for labeling EVs, to characterize and monitor the cellular uptake or tissue distribution of Mca‐EVs. The *in vivo* imaging showed that Dil‐Maca‐EVs efficiently crossed the BBB and had a higher fluorescent density in the mouse brain (Figure 1E,F). Moreover, we demonstrated the efficacy of Maca‐EVs in treating depressed UCMS mice using behavioral tests, such as tail suspension test, forced swim test, open field test, and sucrose consumption test, except for the regulation of sucrose preference (Figure 2).

The gut–brain axis represents bidirectional communication between the central nervous and digestive systems, involving neurotransmitters, neuroimmune, neuroendocrine, and sensory neural pathway [32]. A good deal of data supported the role of the gut–brain axis link in the disease setting of depression [33]. Transplantation of depressed patients' fecal microbiota‐induced depressive‐like behaviors and physiological features in microbiota‐depleted rats, suggesting a causal role of gut microbiota in the development of depression [34]. Additionally, increasing efforts have been devoted to depicting the characteristics of microbiota composition and metabolism in depressive patients. Raes et al. found that butyrate‐producing *Faecalibacterium* and *Coprococcus* bacteria were absent in depression patients [35]. In this study, we found that genus *Lactobacillus* showed a higher abundance in UCMS mice, while the abundance of *Akkermansia*, *Dubosiella*, and *Faecalibaculum* was higher in Control mice than UCMS mice, suggesting their potential role in the antidepressive effects of Maca‐EVs (Figure 3A,B). In addition, the α‐diversity and β‐diversity of the microbiota were varied in the four groups (Figure 3C–E). By regrouping Control, Control + Maca‐EVs, and UCMS + Maca‐EVs into a new group (New_Control), we further demonstrated that Maca‐EVs administration reduced the abundance of *g_Enterococcus* and *g_Lactobacillus*, which were increased in UCMS mice (Figure 3G,H). Most human gut microbiota analysis identified that the abundance of *g_Enterococcus* and *g_Lactobacillus* were depleted in depressive patients [36]. Nevertheless, their abundance in depressed mice failed to reach an agreement. The abundance of *g_Enterococcus* and *g_Lactobacillus* was found to increase in Bharwani's and study [37], but decrease in the studies of Farshim et al. [38] and Galley et al. [39]. It must be admitted that there were inconsistences in the abundance, α‐diversity, and β‐diversity of gut microbiota composition due to individual and species differences [36]. Yang et al. described notably elevated *g*_*Bacteroides* and declined *g_Blautia* and *g_Eubacterium* with altered γ‐aminobutyrate, phenylalanine, and tryptophan metabolism in patients with the major depressive disorder [40]. Here, the Pathway enrichment analysis indicated that UCMS major alterations in metabolic pathway were mapped to biotin, pyrimidine, tyrosine, alanine, aspartate, and glutamate metabolism (Figures 4F and S3E). Moreover, the level of Tyrosine metabolism‐related product l‐5‐hydroxytryptophan was declined in UCMS mice but increased by Maca‐EVs administration (Figure 4C). These suggest that Maca‐EVs increase serum 5‐HT levels through modulation of gut microbiota composition and metabolism.

Excitatory or inhibitory neurotransmitters play an essential role in the homeostatic regulation of neuronal excitability, conferring normal brain functions. The theory that lowered excitatory neurotransmitter 5‐HT levels or activity are associated with depression remains influential. Recently, Moncrieff et al. implied that reduced 5‐HT levels or activity might not be the cause of depression [41]. Nevertheless, currently prescribed drugs for treating depression mainly function by elevating excitatory neurotransmitters 5‐HT, NE, and DA levels throughout the body. Rizzo et al. demonstrated that selective serotonin reuptake inhibitor fluoxetine enhanced 5‐HT expressions, promoting neural stem cell proliferation, which was reversed by 5‐HT receptor antagonism [42]. In our study, all doses of Maca‐EVs significantly increased serum 5‐HT levels in the UCMS mice (Figure 5B). Innate and adaptive immune dysfunctions have been demonstrated to be involved in the pathophysiology of depression [43]. Grosse et al. determined that monocyte gene expression was age related in major depressive disorder patients [44]. Thus, we investigated whether Maca‐EVs would modulate the inflammatory responses by examining the three specific serum inflammatory cytokines, IL‐1β, IL‐6, and TNF‐α, which have been proved to be positively associated with depression [45]. In our model, increased serum IL‐1β, IL‐6, and TNF‐α were detected in the UCMS mice, but were not statistically significant (Figure 5C–E). This may be because we tested the inflammatory factors at a late phase of depression induction. Nevertheless, Maca‐EVs significantly decreased the IL‐6 and TNF‐α levels only at a dose of 200 μg/kg. These data suggested that the 5‐HT signaling is more sensitive to the Maca‐EVs treatment, and we next focused on its action in treating depressive UCMS mice in this study.

There are synergistic effects between 5‐HT and BDNF in modulating synaptic plasticity that 5‐HT triggers the expressions of BDNF, and BDNF improves 5‐HT neuronal growth and survival and maintains synaptic plasticity in the adult brain [21]. The activation of 5‐HT signaling requires the interaction with 5‐HT receptors that is not only coupled with but also activates the G‐protein (Rho, Rac, and Cdc42)‐related pathway [46]. Udo et al. demonstrated that the 5‐HT activated GTP‐Cdc42 in aplysia sensory neurons, promoting learning‐related synaptic growth [47]. Elena et al. identified that the activation of the 5‐HT receptor leads to filopodia formation via a Cdc42‐mediated pathway accompanied with RhoA relevant cell rounding in neuroblastoma cells [48]. Errico et al. [49] and Speranza et al. [50] showed that the 5‐HT signaling activation enhanced neuron function via the ERK pathway. Others also identified that (R)‐ketamine isomer exerts antidepressant effects via activating the ERK‐NRBP1‐CREB‐BDNF pathway [51, 52]. Indeed, elevated ratios of GTP‐Cdc42/Cdc42 and p‐ERK/ERK, and BDNF expression were noted in the cortex and hippocampus of Maca‐EVs‐injected UCMS mice (Figures 6A–C and S4A–C). Evidence has suggested that BDNF signaling via its receptor TrkB is demanded for the effects of antidepressant drugs. Adachi et al. found that the absence of TrkB but not the deletion of BDNF caused decreased capacity in treating depression [53]. He et al. showed that the *Alpinia oxyphylla* Miq. Hippocampal neurogenesis was promoted via activating BDNF/TrkB/AKT signaling pathway [54]. Accordingly, we also found that the Maca‐EVs increased the expression of TrkB and phosphorylated AKT in the cortex and hippocampus of UCMS mice (Figures 6D and S4D,E). The *in vitro* assay further confirmed that 5‐HT enhanced BDNF expression and consequent stimulation of TrkB/AKT signaling via the GTP‐Cdc42/ERK pathway (Figure 6F–J).

Unfortunately, we only initially isolated Maca‐EVs and investigated the antidepressant effect. We observed that Maca‐EVs could influence the fecal microbiota composition and elevate serum 5‐HT levels, leading to increased BDNF expression via activation of the GTP‐Cdc42/ERK signaling pathway. Enterochromaffin cells of the intestinal mucosa are the primary site for the 5‐HT synthesis, storage, and release. We revealed a potential role of Maca‐EVs in modulating fecal microbiota/metabolite to produce 5‐HT, which requires further experiments to clarify. However, the EVs could cross the BBB and they may exert their antidepressant therapeutic activities by directly affecting the brain or through the peripheral pathway. In this study, we only focused on the effects of Maca‐EVs on modulating the gut–brain axis. The administration of Maca‐EVs via gavage or targeted delivery to the brain would be employed in future studies to elucidate the underlying mechanisms of Maca‐EVs in treating depression.

There are several other limitations in the present study. First, Maca‐EVs produce antidepressant‐like effects similar to those of Maca or its extract, possibly because Maca‐EVs contain them. Notably, the nanoscale size of Maca‐EVs (<200 nm) allows them to overcome the disadvantages of Maca extract components, like, polysaccharides, proteins, and alkaloids (which are high molecular and insoluble), thereby enabling them to cross the BBB or intestinal barrier. Nevertheless, thorough omics studies should be performed to understand the biologically active constituents, such as nucleic acids, proteins, lipids, and secondary metabolites, to validate this speculation. Besides, the key elements that confer the antidepressant mechanism of Maca‐EVs remain unexplored. Recently, EVs containing miRNAs have attracted enormous interest in commercial spans as next‐generation medicine [55]. However, a single miRNA may not be as effective as multiple miRNAs. Occasionally, even though some miRNAs reveal excellent therapeutic capacity, it is challenging to develop into drugs due to their ease of degradation, multiple targets, and the potential biological safety risk. For instance, the Phase 1 clinical trial (NCT02862145) on evaluating MRX34's (miRNA‐34) efficacy in treating melanoma was withdrawn due to severe immune‐related side events. Emerging studies pointed out that the EVs' therapeutic activity originated from the synergetic effects of their contents [56]. Thus, it appears to be less important to identify the precise element that plays an essential role in the function of EVs. Second, we only unraveled the role of 5‐HT/BDNF in the antidepressant activity of Maca‐EVs. Apart from the BDNF, other neurotrophic factors including vascular endothelial growth factor, fibroblast growth factor 2, insulin‐like growth factor 1, and Activin‐A are also of interest to study. The mechanisms involved in two other elevated monoamine neurotransmitters in antioxidant or anti‐inflammatory effects require further investigation. Lastly, a sucrose test that may affect the composition of intestinal flora was conducted to assess the depression behavior, though our data showed the Maca‐EVs did not influence the sucrose preference level. In the future study, we will avoid these technical points in investigating the exact mechanism of Maca‐EVs in modulating the gut‐grain axis and their associations with Maca‐EVs' therapeutics in treating depression.

## CONCLUSION

Taken together, this study reports for the first time the successful isolation and characterization of Maca‐EVs, which exhibit antidepressant effects in UCMS mice. These effects were accompanied by alteration in depression‐related fecal microbiota and enhanced gut 5‐HT metabolism, as well as elevated serum 5‐HT concentrations. We demonstrated that Maca‐EVs enhance the 5‐HT‐regulated BDNF expressions by activating the BDNF/TrkB/AKT axis. These data suggest that the regulation of the gut–brain axis by Maca‐EVs may be one element of its therapeutic mechanisms. Given the ability of PDEVs to cross the BBB, Maca‐EVs represent a promising platform for the management of depression. However, further studies are required to fully evaluate the safety and efficacy of this approach before clinical application.

## AUTHOR CONTRIBUTIONS

Su‐Hua Qi and Bing Gu conceived and designed this study. Rui Hong, Lan Luo, Liang Wang, and Zhao‐Li Hu performed animal experiments and/or analyzed data and/or prepared the figures. Rong‐Qi Yin, Ming Li, Bin Gu, and Bin Wang analyzed data. Tao Zhuang and Xin‐Yue Zhang performed animal experiments. Wan Wang, Yuan Zhou, and Lin‐Yan Huang assisted the study. Rui Hong, Lan Luo, and Liang Wang wrote the first version of the manuscript with input from coauthors. Su‐Hua Qi and Bing Gu revised the manuscript. All authors have read and approved the final manuscript.

## CONFLICT OF INTEREST STATEMENT

The authors declare no conflict of interest.

## ETHICS STATEMENT

The ethics application (No. 202006W037) was approved by the Research Ethics Committee of Xuzhou Medical University.

## Supporting information

Supporting information.

## Data Availability

A data availability statement confirms the presence or absence of shared data. All data generated or analyzed during this study are included in this article and its supplementary files. Supplementary materials (figures, tables, scripts, graphical abstract, slides, videos, Chinese translated version, and update materials) may be found in the online DOI or iMeta Science http://www.imeta.science/.
