# Correction to “*Lepidium meyenii* Walp (Maca)‐derived extracellular vesicles ameliorate depression by promoting 5‐HT synthesis via the modulation of gut–brain axis”

**DOI:** 10.1002/imt2.259

**Published:** 2024-12-15

In Hong et al. [1], following details should have been corrected.

1. In page 6, “We also investigated the effect of Maca‐EVs on microbial diversity and found that microbial richness as indicated by the Faith‐pd and Observed features index was higher in the Maca‐EVs groups (Control+Maca‐EVs and UCMS+Maca‐EVs).” was incorrect.

This should be: “We also investigated the effect of Maca‐EVs on microbial diversity and found that microbial richness as indicated by the Faith‐pd and Observed features index was lower in the Maca‐EVs groups (Control+Maca‐EVs and UCMS+Maca‐EVs).”

2. In page 6, the sentence “Only the Faith pd index in Control + Maca EVs group was significantly lower than that in the Control group (*p* < 0.05, figure 3D).” should be deleted.

3. In Figure 4, page 8 of the article, “(F) Reciprocal interactions between altered gut bacteria and serum metabolites identified by a co‐occurrence network based on Spearman correlation analysis in pos mode.” was incorrect.

This should be: “(F) Reciprocal interactions between altered gut bacteria and metabolites identified by a co‐occurrence network based on Spearman correlation analysis in pos mode.”

We apologize for these errors.

## References

[1] Hong, Rui , Lan Luo , Liang Wang , Zhao‐Li Hu , Qi‐Rong Yin , Ming Li , Bin Gu , et al. 2023. “ *Lepidium meyenii* Walp (*Maca*)‐Derived Extracellular Vesicles Ameliorate Depression by Promoting 5‐HT Synthesis via the Modulation of Gut–Brain Axis.” iMeta e116. 10.1002/imt2.116 38867934 PMC10989901

