# Supplementary material for: Lepidium meyenii Walp (Maca)‐derived extracellular vesicles ameliorate depression by promoting 5‐HT synthesis via the modulation of gut–brain axis
Source: Imeta. 2023 Jun 4;2(3):e116. doi: 10.1002/imt2.116 (PMC10989901; doi:10.1002/imt2.116)

***Lepidium meyenii* Walp (Maca)-derived extracellular vesicles ameliorate depression by promoting 5-HT synthesis via the modulation of gut-brain axis**

Running title: Antidepressant effects of Maca-EVs

Rui Hong^1,2#^, Lan Luo^1#^, Liang Wang^3,4,5#^, Zhao-Li Hu^6#^, Qi-Rong Yin^2,7^, Ming Li^1^, Bin Gu^6^, Bin Wang^7^, Tao Zhuang^2^, Xin-Yue Zhang^2^, Yuan Zhou^1^, Wan Wang^1^, Lin-Yan Huang^1^, Bing Gu^1,3^*, Su-Hua Qi^1,2^*

^1^School of Medical Technology, Xuzhou Medical University, Tongshan Road 209, Xuzhou 221004, China

^2^School of Pharmacy, Xuzhou Medical University, Tongshan Road 209, Xuzhou 221004, China

^3^Laboratory Medicine, Guangdong Provincial People's Hospital, Guangdong Academy of Medical Sciences, Southern Medical University, Zhongshan Road 106, Guangzhou 510080, China

^4^School of Medical Informatics and Engineering, Xuzhou Medical University, Tongshan Road 209, Xuzhou 221004, China

^5^Centre for Precision Health, School of Medical and Health Sciences, Edith Cowan University, Perth, Western Australia 6027, Australia

^6^Research Center for Biochemistry and Molecular Biology and Jiangsu Key Laboratory of Brain Disease Bioinformation, Xuzhou Medical University, Tongshan Road 209, Xuzhou 221004, China

^7^Department of Laboratory Medicine, Affiliated Hospital of Xuzhou Medical University, Huaihai West Road 99, Xuzhou 221004, China.

^#^These authors contributed equally: Rui Hong, Lan Luo, Liang Wang, Zhao-Li Hu

*Correspondence: [suhuaqi@xzhmu.edu.cn](mailto:suhuaqi@xzhmu.edu.cn) (Su-Hua Qi), gubing@gdph.org.cn (Bing Gu)

**Supplementary Materials and Methods**

**Animals**

Male C57BL/6J mice weighing 18-22 g were purchased from the Beijing Vital River Laboratory Animal Technology Co., Ltd. This study was approved by the Institutional Animal Care and Use Committee of Xuzhou Medical University (202006W037). All animal procedures were performed following the institutional and national guidelines.

**Isolation of Maca-EVs**

Fresh *Lepidium meyennii* Walp (Lijiang City, Yunnan Province, China) were carefully washed three times with deionized water and squeezed into juice. The juice was centrifuged at 1000 × g for 10 min, 3000 × g for 20 min, and 10 000 × g for 40 min at 4 °C. Then the supernatant was ultracentrifuged at 150 000 × g for 90 min (Optima XE-90, Beckman Coulter Life Sciences, Indianapolis, U.S.). The pellets were resuspended in phosphate buffered saline (PBS) and transferred to a gradient sucrose solution (8%, 30%, 45%, and 60%) with subsequent ultracentrifugation at 150 000 × g for 90 min. The band between 30% and 45% sucrose layer was collected and washed with PBS. Then it was ultracentrifuged at 150 000 × g for 90 min and passed through with a 0.22 μm filter (#SLGV004SL, Millipore) to obtain sterile Maca-EVs. The BCA assay kit (#P001, Beyotime) was used for the protein quantification of Maca-EVs.

**Transmission electron microscopy**

Maca-EVs (10 μl) was adsorbed to a carbon-coated grid for 1 min. Then it was washed with one drop water and stained for 15 s with 1% uranyl acetate. Absorbed Maca-EVs were examined using a transmission electron microscopy (FEI Tecnai G2 Spirit Twin, New York, USA).

**Nanoparticle tracking analysis**

The size distribution of Maca-EVs was examined by a nanoparticle tracking analyzer (ZetaView, Bavaria, Germany). According to the manual, the Maca-EVs were diluted with PBS and then added to the analytical cell to initiate the assay. The data of size distribution were obtained.

***In vivo* uptake of Maca-EVs**

The isolated Maca-EVs were incubated with lipophilic ﬂuorescent dye Dil (Molecular Probes, Oregon, USA) for 30 min at room temperature. The Dil-labeled Maca-EVs (Maca-EVs-Dil) were then ultracentrifuged at 150 000 × g for 90 min. Then the supernatant was discarded and resuspended with 200 μl PBS. C57BL/6J mice were intravenously administrated with Maca-EVs-Dil or free-Dil (200 μg/kg), and then imaged by the IVIS® Spectrum system (Perkin Elmer, Massachusetts, USA) at 12 h.

**Unpredictable chronic mild stress (UCMS) model and Drug administration**

The UCMS model and drug administration design were established according to the previous reports [1, 2]. The UCMS model was established by subjecting daily 2 or 3 types of mild unpredictable stressors listed below randomly to the C57BL/6J mice for 8 weeks as reported. There are 12 types of stressors, including clip tail for 5 min, cold swim at 0 °C for 5 min, 45 ^o^ titled cage for 12 h, restraint for 6h, food or water deprivation for 12 h, damp cage for 24 h, empty cage for 12 h, high speed vibration for 4 h, flash lamp irradiation for 12 h, soiled cage bedding for 6 h, inversion of day/night light cycle for 24 h, cat sounds, hair for 4h. Each stressor was not applied consecutively. The control and UCMS mice were housed separately. The mice body weight was weighed every two days until sacrificed.

Subsequently, the UCMS mice were [intravenous](C:/Users/JMC/AppData/Local/youdao/dict/Application/8.10.3.0/resultui/html/index.html#/javascript:;)ly administrated with Maca-EVs (0, 50, 100, 200 μg/kg) once every 2 days for 2 weeks. Importantly, UCMS model were maintained daily during the two weeks of Maca-EVs treatment, avoiding potential recovery from UCMS due to the high self-repair capability of mice. Stress free mice that injected with Maca-EVs (0, 100 μg/kg) were included as control. Last, mice were sacrificed to obtain fecal pellets, blood sample, and brain tissues after the behavioral tests were accomplished. Each mouse was separately kept in a sterilized box to collect fresh fecal pellets. Fecal samples of 200 mg were snap frozen and stored at -80 °C until processing. The serum was collected by centrifugation the blood sample at 3500 rpm for 10-15 min at 4 °C. Then serum was transferred to a fresh Eppendorf tube and stored at -80 °C for further analysis. Fresh brain tissues were fixed in 4% paraformaldehyde at 4 °C overnight. Then it was immersed in 15% and 30% sucrose solution for complete dehydration and embedded in OCT compound (#4583 Tissue-Tek® O.C.T. Compound, Sakura) to prepare frozen sections (14 μm). Fresh brain tissues were also kept in an ice-cold saline solution. The hippocampus and cortex tissues were isolated on a cold dish and frozen in liquid nitrogen immediately and stored at -80 °C for western blot assay. The hippocampus and cortex were also homogenized with ice-cold RIPA buffer (#89900, Thermo Scientific) containing 1% phosphatase and protease inhibitors (#78442, Thermo Scientific), respectively. Then centrifuged at 16, 000×g at 4°C for 15 min, and the obtained supernatants were used for assay.

**Tail suspension test (TST)**

The mice were suspended upside down with tails fixed on the bracket using adhesive tape in the TST-100 tail suspension chamber (Tai-Co software, Chengdu, China). After 1 min of adaptation, Tail Suspension Scan TM (Clever Sys Inc., VA, USA) was used to record and analyze the immobility time of the mice over the subsequent 5 min (activity threshold value = 30%) [2].

**Forced swimming test (FST)**

Mice were separately placed in a glass cylinder (diameter, 10 cm, Tai League Software, Chengdu, China) filled with water (25 ± 1°C) to a depth of 14 cm for 6 min. The duration of immobility was recorded and analyzed during the last 5 min using Tail Suspension Scan TM (Clever Sys Inc., VA, USA). Mice were regarded as immobile when they gave up escaping and kept their heads above water [2].

**Open field test (OFT)**

Each mouse was carefully placed in the center of an open field chamber (62.5 × 74 × 51 cm), and behaviors were recorded for 5 min by a video camera. The data of total travelling distance and entries in the zone were analyzed using an OFT software (SMART 3.0, Panla, Spain) [3].

**Novelty suppressed feeding test (NSFT)**

The NSFT was carried out as previously described [4]. Mice were deprived of food for 24 h before being placed in a white plastic box (50 cm × 50 cm × 20 cm) whose floor was covered with wooden bedding (changed for each mouse). A single food pellet (regular chow) was placed on a piece of filter paper (10 cm in diameter), positioned in the center of the container that was brightly illuminated (~500 lux). The mouse was placed in one corner of the box and the latency to eat was measured during 10 min. Feeding was defined as biting, not simply sniffing or touching the food. Immediately after the test, the animals were transferred into their home cage.

**Splash test (ST)**

The splash test was performed by spraying 10% sucrose solution on the dorsal coat of a mice in its home cage. The sucrose solution dirties the coat and increases latency to groom. The time spent grooming of mice was recorded for a period of 6 min [5].

**Sucrose preference test (SPT)**

After a 24-hour period of water deprivation, each mouse was exposed to two bottles for 12 h, one containing deionized water and another containing 1% sucrose solution. The positions of two bottles were placed randomly and exchanged after 6 h to avoid possible side preference. The sucrose preference percentage was calculated by the ratio of the sucrose solution consumption vs. the total liquid consumption [2].

**Elevated-plus maze (EPM) test**

This EPM test is employed to assess anxiety-related behaviors in rodents [6]. The EPM apparatus comprises a “+” shape mase above the floor with two open arms (OA) and two closed arms (CA), connected by a central platform. Mice were placed in the center of the maze and behavior was recorded by means of a video camera mounted above the maze and analyzed using a video tracking system. The time for being in open arms or closed arms is scored to measure anxiety-like behavior.

**16S rRNA sequencing**

Total genome DNA from fecal pellets was extracted using CTAB/SDS method. DNA concentration and purity was monitored on 1% agarose gels. According to the concentration, DNA was diluted to 1ng/µl using sterile water. 16S rRNA/18SrRNA/ITS genes of distinct regions (16S V4/16S V3/16S V3-V4/16S V4-V5, 18S V4/18S V9, ITS1/ITS2, Arc V4) were amplified used specific primer (e.g. 16S V4:515F-806R, 18S V4: 528F-706R, 18S V9: 1380F-1510R, et. al) with the barcode. All PCR reactions were carried out with 15 µl of Phusion® High-Fidelity PCR Master Mix (New England Biolabs), 0.2 µM of forward and reverse primers, and about 10 ng template DNA. Thermal cycling consisted of initial denaturation at 98 °C for 1 min, followed by 30 cycles of denaturation at 98 °C for 10 s, annealing at 50 °C for 30 s, and elongation at 72 °C for 30 s, finally 72 °C for 5 min. Mix same volume of 1X loading buffer (contained SYB green) with PCR products and operate electrophoresis on 2% agarose gel for detection. PCR products was mixed in equal density ratios. Then, mixture PCR products was purified with Qiagen Gel Extraction Kit (Qiagen, Germany). Sequencing libraries were generated using TruSeq® DNA PCR-Free Sample Preparation Kit (Illumina, USA) following manufacturer's recommendations and index codes were added. The library quality was assessed on the Qubit@ 2.0 Fluorometer (Thermo Scientific) and Agilent Bioanalyzer 2100 system. At last, the library was sequenced on an Illumina NovaSeq platform and 250 bp paired-end reads were generated.

Statistical analyses were performed using the statistical software R (R version R-4.1.2), Python (Python 3.8.12 version), and Ubuntu (Ubuntu release 18.04) when data were not normally distributed, normal transformations were attempted using of area normalization method.

**Untargeted Metabolomics**

The samples were placed in the EP tubes and resuspended with prechilled 80% methanol by well vortex. Then the samples were melted on ice and whirled for 30 s. After the sonification for 6 min, they were centrifuged at 5 000 rpm, 4°C for 1 min. The supernatant was freeze-dried and dissolved with 10% methanol. Finally, the solution was injected into the LC-MS/MS system analysis.

UHPLC-MS/MS analyses were performed using a Vanquish UHPLC system (ThermoFisher, Germany) coupled with an Orbitrap Q Exactive^TM^ HF mass spectrometer (Thermo Fisher, Germany) in Novogene Co., Ltd. (Beijing, China). Samples were injected onto a Hypesil Goldcolumn (100 × 2.1 mm, 1.9 μm) using a 17-min linear gradient at a flow rate of 0.2 mL/min. The eluents for the positive polarity mode were eluent A (0.1% FA in Water) and eluent B (Methanol). The eluents for the negative polarity mode were eluent A (5 mM ammonium acetate, pH 9.0) and eluent B (Methanol).The solvent gradient was set as follows: 2% B, 1.5 min; 2-100% B, 3 min; 100% B, 10 min; 100-2% B, 10.1 min; 2% B, 12 min. Q Exactive^TM^ HF mass spectrometer was operated in positive/negative polarity mode with spray voltage of 3.5 kV, capillary temperature of 320°C, sheath gas flow rate of 35 psi and aux gas flow rate of 10 L/min, S-lens RF level of 60, Aux gas heater temperature of 350 °C.

The raw data files generated by UHPLC-MS/MS were processed using the Compound Discoverer 3.1 (CD3.1, ThermoFisher) to perform peak alignment, peak picking, and quantitation for each metabolite. The main parameters were set as follows: retention time tolerance, 0.2 minutes; actual mass tolerance, 5ppm; signal intensity tolerance, 30%; signal/noise ratio, 3; and minimum intensity, et al. After that, peak intensities were normalized to the total spectral intensity. The normalized data was used to predict the molecular formula based on additive ions, molecular ion peaks and fragment ions. And then peaks were matched with the mzCloud (https://www.mzcloud.org/), mzVault and MassList database to obtain the accurate qualitative and relative quantitative results.

Statistical analyses were performed using the statistical software R (R version R-4.1.2), Python (Python 3.8.12 version), and Ubuntu (Ubuntu release 18.04) when data were not normally distributed, normal transformations were attempted using of area normalization method.

**UPLC-MS/MS**

Serum samples (10 μl) were precipitated with 40 μl methanol, then vortexed at 2 000 rpm for 3 min and centrifuged at 13 000 rpm for 5 min at 4 °C. Then, supernatant (40 μl) was loaded on the machine. Standard stock solution was diluted with methanol and prepared a series concentration of standard solution (0.1, 0.2, 0.5, 1, 2, 5, 10, 20, 50, 100, 200, 300) the unit for 5-HT is ng/mL, for NE and DA is pg/mL.

**Enzyme-linked immunosorbent assay (ELISA)**

Serum inflammatory factors IL-6 (#MM-0163M2), IL-1β (#MM-0040M2), and TNF-α (#MM-0132M2) were determined by ELISA (MEIMIAN, China) according to the manual. The absorbance value was measured by Multiskan^TM^ FC (Thermo Fisher Scientific) at 450 nm. The concentration was calculated based on a standard curve equation.

**Cell culture**

PC12 cells (#CRL-1721™, ATCC) were grown in DMEM (Hyclone, #SH30021.01, Logan,Vtah), supplemented with 10% fetal bovine serum (FBS), 100 U/mL penicillin, and 0.1 mg/mL streptomycin (#C0222, Beyotime). Cells were maintained in a humidified incubator with a 95% air/5% CO2 atmosphere at 37 °C. The cells were separately treated with 5-HT (5 μM) or ML141 (5 mM) for 12 h and cultured for another 24 h. Cells with no other treatments were included as control.

**Measurement of Cdc42 GTPase activity**

Cdc42 GTPase activity was performed using the Rac1/Cdc42 activation assay kit (#17–441, Sigma-Aldrich). Briefly, cells were lysed with 300 µl modified lysis buffer. Add Rac/cdc42 Assay Reagent directly to the lysate immediately after removing cellular debris and insoluble material by centrifugation. Gently rock the reaction mixture at 4°C for 60 min. Collect the agarose beads by pulsing (5 s in the microcentrifuge at 14 000 g) and drain off the supernatant. Wash the beads 3 times with MLB. Resuspend the agarose beads in an appropriate amount of 2×Laemmli sample buffer and boil for 5 min. Use 20 µl per assay for 10 × 10 cm mini gels. The supernatant was collected for SDS-PAGE and subsequent immunoblot analysis. Probe the blot with anti-Cdc42, followed by HRP-conjugated secondary antibody and ECL reagent.

**Western Blotting**

After indicated treatments, PC12 cells were homogenized with ice-cold RIPA buffer (#89900, Thermo Scientific) containing 1% phosphatase and protease inhibitors (#78442, Thermo Scientific). Total protein concentration was measured by BCA assay kit (#P001, Beyotime). The proteins were separated on SDS-PAGE gels (#KGP113K, Key GEN Bio Tech) and transferred to 0.22 μm PVDF membranes (#R1JB38277, Millipore) via Trans-Blot®SD cell (#221BR5621). Membranes were blocked with 5% BSA in Tris Buffered Saline with Tween 20 (TBST) for 1 h at RT and then incubated with the primary antibody at 4°C overnight. The primary antibodies included CD63 (1:500, #25682-1-AP, Proteintech), TSG101 (1:2000, #28283-1-AP, Proteintech), Cdc42 (1:10000, #ab187643, Abcam), BDNF (1:1000, #ab108319, Abcam), TrkB (1:1000, #4603, CST), AKT (1:1000, #AF626, Proteintech), p-AKT (1:2000, #4060, CST), ERK (1:1000, #BF8004, Proteintech), p-ERK (1:1000, #AF1015, Proteintech), and α-tubulin (1:5000, #66031-1-Ig, Proteintech). After sufficient washing with TBST, membranes were incubated with appropriate HRP-conjugated secondary antibodies for 1 h at RT. Blots were visualized using an ECL detection kit (Millipore, #WBKLS0500) using a ChemiDocTM Touch imaging system (Bio-Rad, California, U.S.). The density of the band was analyzed by the Image lab software (Bio-Rad, California, U.S.).

**Immunofluorescence staining**

The brain cryosections or PC12 cells slices with dedicated treatments were blocked with 5% BSA with 0.3% Triton (#XK0193, xuanke, China) for 1 h at RT. Then sections were stained with primary antibodies BDNF (1:200, #ab187643, Abcam) and NeuN (1:500, #12943, CST), the PC12 cells slices were stained with BDNF (1:200, #ab187643, Abcam) overnight at 4°C. Then sections or slices were washed with 1% PBST three times for 15 min. Then sections or slices were stained with anti-mouse 488 (1:800) and anti-rabbit 568 (1:500) for 1.5 h. Nucleus were stained with DAPI (40,60-diamidino-2-phenylindole) for 15 min at RT. Cells were observed under a Leica laser scanning confocal microscope (STELLARIS 5, Germany).

**Statistical analysis**

All values are presented as the mean ± SEM. The statistical significance between two groups was performed by unpaired student’s t test (assume both populations have the same SD) or with Welch’s correction (do not assume equal SDs) when data follow a normal distribution. If not, the statistical significance between two groups was performed by Mann-Whitney U test. The statistical significance between three or more groups was performed by the ordinary one-way analysis of variance (ANOVA) followed by the Tukey’s multiple comparisons test (assume all populations have the same SD) or Brown-Forsythe and Welch ANOVA tests followed by the Tamhane T2 multiple comparisons test (do not assume equal SDs) when data follow a normal distribution. If not, the statistical significance between three or more groups was performed by Kruskal-Wallis test followed by Dunn’s multiple comparisons test. The statistical significance evaluation of repeat measurement of mice body weight was performed by RM two-way ANOVA followed by the Tukey’s multiple comparisons test (GraphPad Prism 9.3.0). Differences were considered significant when *p* < 0.05.

**Supplementary REFERENCES**

1. Chevalier, Grégoire, Eleni Siopi, Laure Guenin-Macé, Maud Pascal, Thomas Laval, Aline Rifflet, Ivo Gomperts Boneca, et al. 2020. “Effect of gut microbiota on depressive-like behaviors in mice is mediated by the endocannabinoid system.” *Nature Communications* 11: 6363. <https://doi.org/10.1038/s41467-020-19931-2>

2. Shu, Xiaodong, Yiming Sun, Xiyang Sun, Yuanzhang Zhou, Yaqi Bian, Zhaoma Shu, Jianhua Ding, Ming Lu, Gang Hu. 2019. “The effect of fluoxetine on astrocyte autophagy flux and injured mitochondria clearance in a mouse model of depression.” *Cell Death & Disease* 10: 577. <https://doi.org/10.1038/s41419-019-1813-9>

3. Taniguti, E. H., Y. S. Ferreira, I. J. V. Stupp, E. B. Fraga-Junior, D. L. Doneda, L. Lopes, F. Rios-Santos, et al. 2019. “Atorvastatin prevents lipopolysaccharide-induced depressive-like behaviour in mice.” *Brain Research Bulletin* 146: 279-286. <https://doi.org/https://doi.org/10.1016/j.brainresbull.2019.01.018>

4. Blasco-Serra, Arantxa, Eva M. González-Soler, Ana Cervera-Ferri, Vicent Teruel-Martí, Alfonso A. Valverde-Navarro. 2017. “A standardization of the Novelty-Suppressed Feeding Test protocol in rats.” *Neuroscience Letters* 658: 73-78. <https://doi.org/https://doi.org/10.1016/j.neulet.2017.08.019>

5. Hu, Congli, Ying Luo, Hong Wang, Shengnan Kuang, Guojuan Liang, Yang Yang, Shaoshan Mai, Junqing Yang. 2017. “Re-evaluation of the interrelationships among the behavioral tests in rats exposed to chronic unpredictable mild stress.” *PLoS One* 12: e0185129. <https://doi.org/10.1371/journal.pone.0185129>

6. Bahi, Amine, Jean-Luc Dreyer. 2019. “Dopamine transporter (DAT) knockdown in the nucleus accumbens improves anxiety- and depression-related behaviors in adult mice.” *Behavioural Brain Research* 359: 104-115. <https://doi.org/https://doi.org/10.1016/j.bbr.2018.10.028>

**Figure S1. Successful establishment of UCMS model.** (A) The evaluation of body weight on the Control and UCMS mice. Behavior tests on the depression characters of Control and UCMS regarding the tail suspension test (B), forced swimming test (C), splash test (D), novelty suppressed feeding test (E), and sucrose preference test (F). Behavior tests on the anxiety characters of Control and UCMS regarding the Elevated-plus maze test (G, H). All data are presented as mean ± SEM (n=15 to 30 experiments for each group). The statistical significance was performed by unpaired student’s t test in (B), Mann-Whitney U test in (A, C-F), and Kruskal-Wallis test followed by Dunn’s multiple comparisons test in (G, H). ns, not significant, *p* > 0.05, **p* < 0.05, ***p* < 0.01, ****p* < 0.001 between two indicated groups.


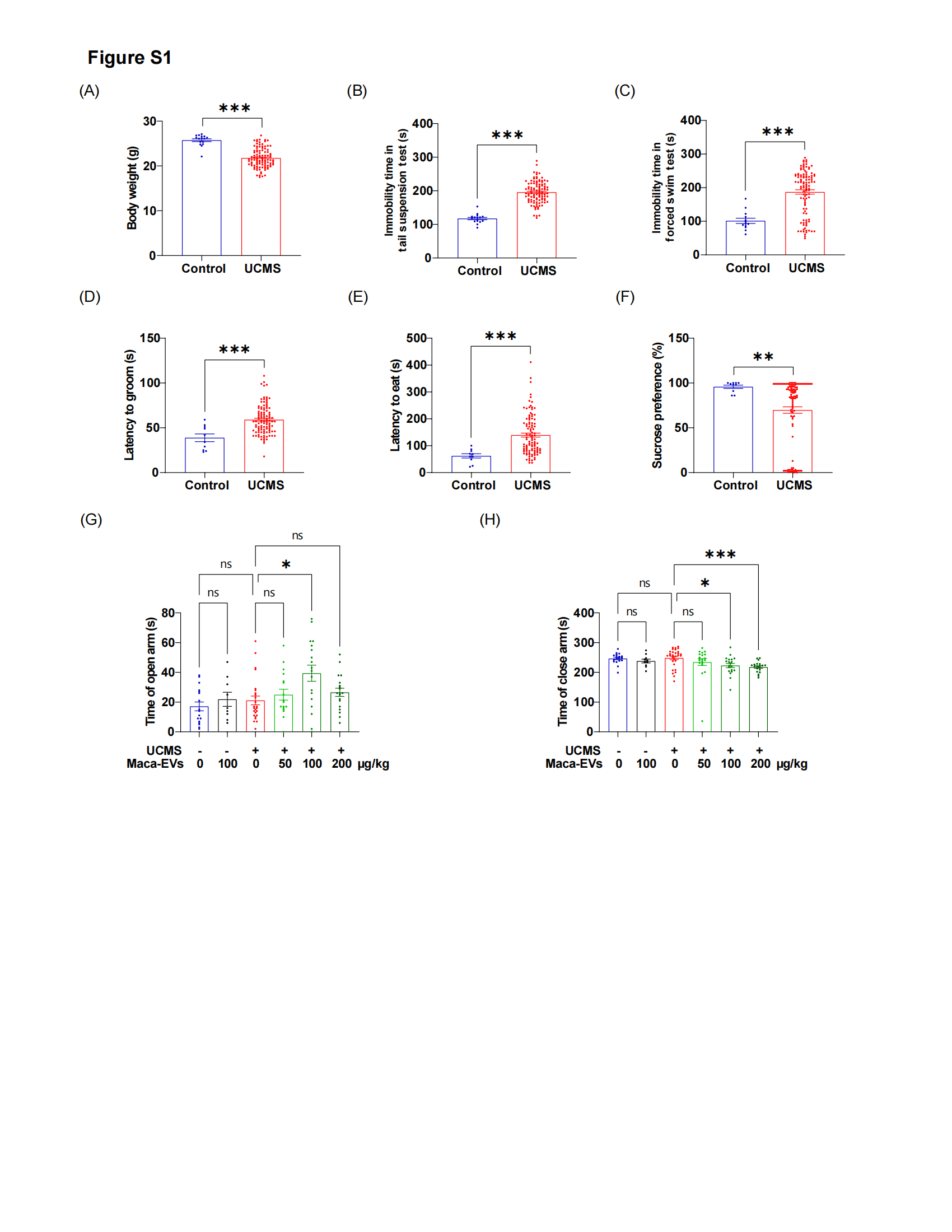


**Figure S2. Microbiota compositions among groups.** (A) Venn diagram on the microbiota compositions in Control, UCMS, Control+Maca-EVs, UCMS+Maca-EVs group. (B) Partial least squares-discriminant analysis (PLS-DA) analysis between sample groups. (C-F) The abundance of typical bacteria that were significantly different between the Control and UCMS groups. Significance was evaluated by ordinary one-way analysis of variance (ANOVA) followed by the Turkey’s multiple comparisons test in (C-F). **p* < 0.05 between two indicated groups.


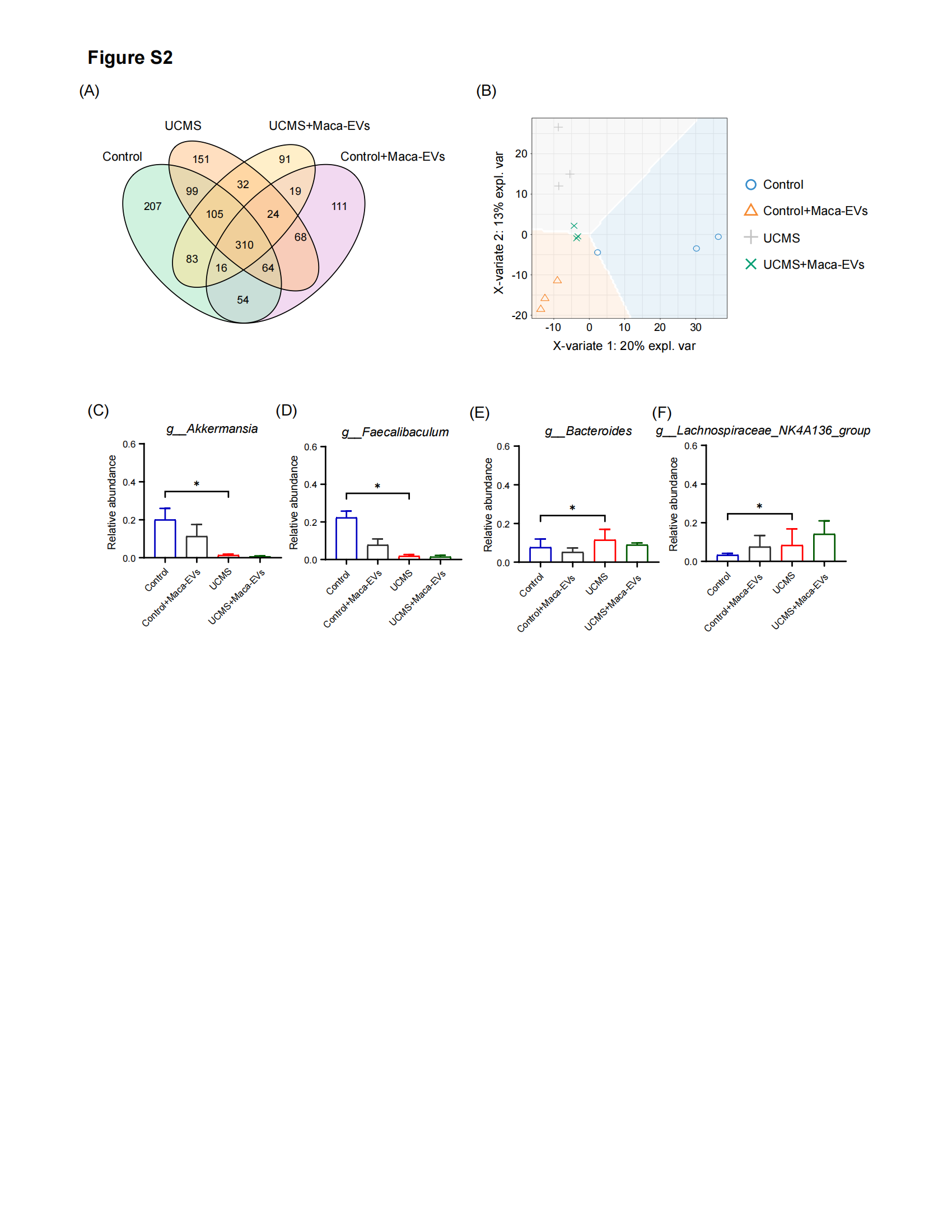

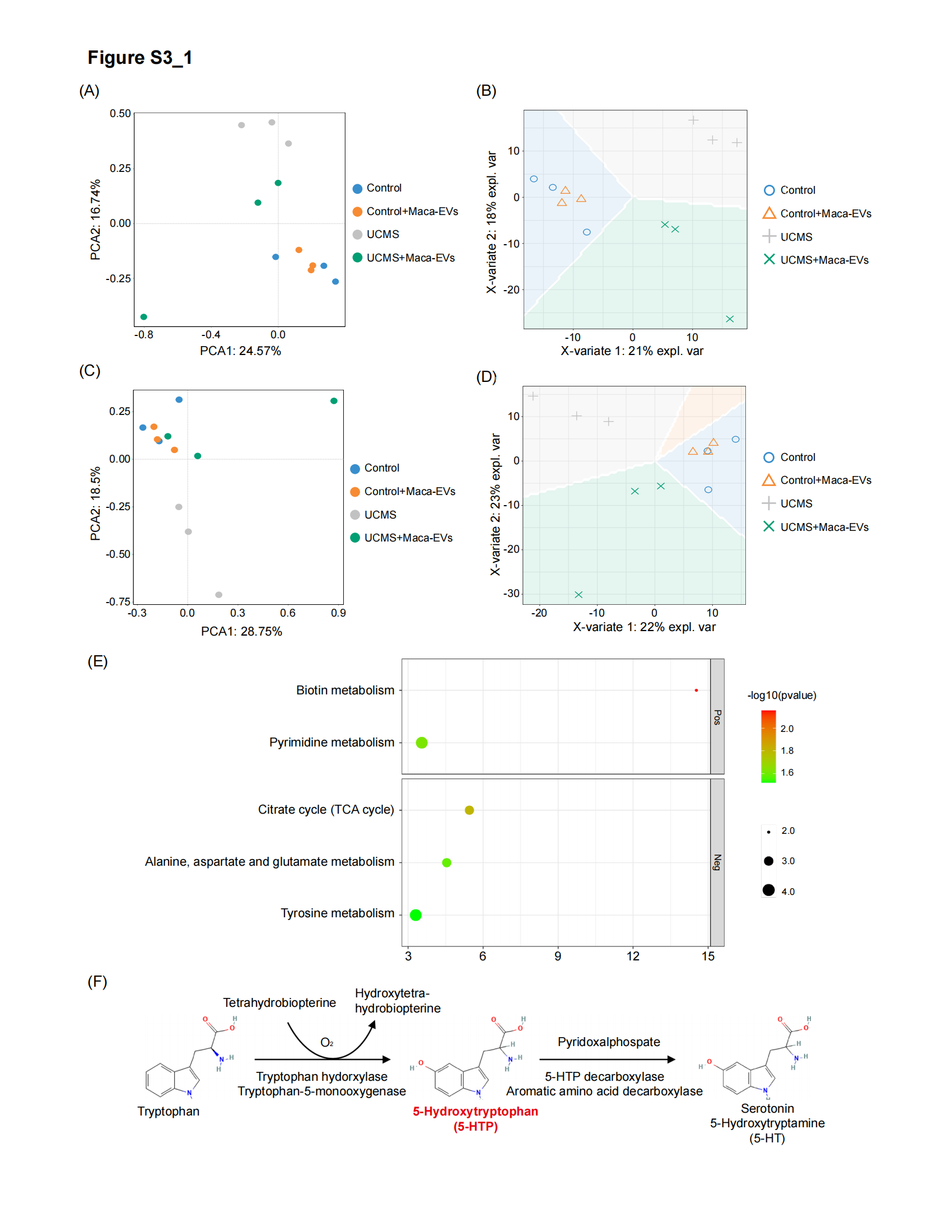


**Figure S3. Metabolomics changes among groups.** Principal components analysis (PCA) and partial least squares-discriminant analysis (PLS-DA) on the fecal metabolic signatures in pos (A, B) and neg (C, D) mode. (E) Pathway enrichment analysis on the UCMS major alterations in metabolic pathways. (F) Representative image showing the metabolic pathway of 5-HT. (G) Differential metabolites between New_Control and UCMS group (VIP > 1, *p* < 0.05, |logFC| > 1) in neg mode. (H) Reciprocal interactions between altered gut bacteria and serum metabolites identified by a co-occurrence network based on Spearman correlation analysis in neg mode.


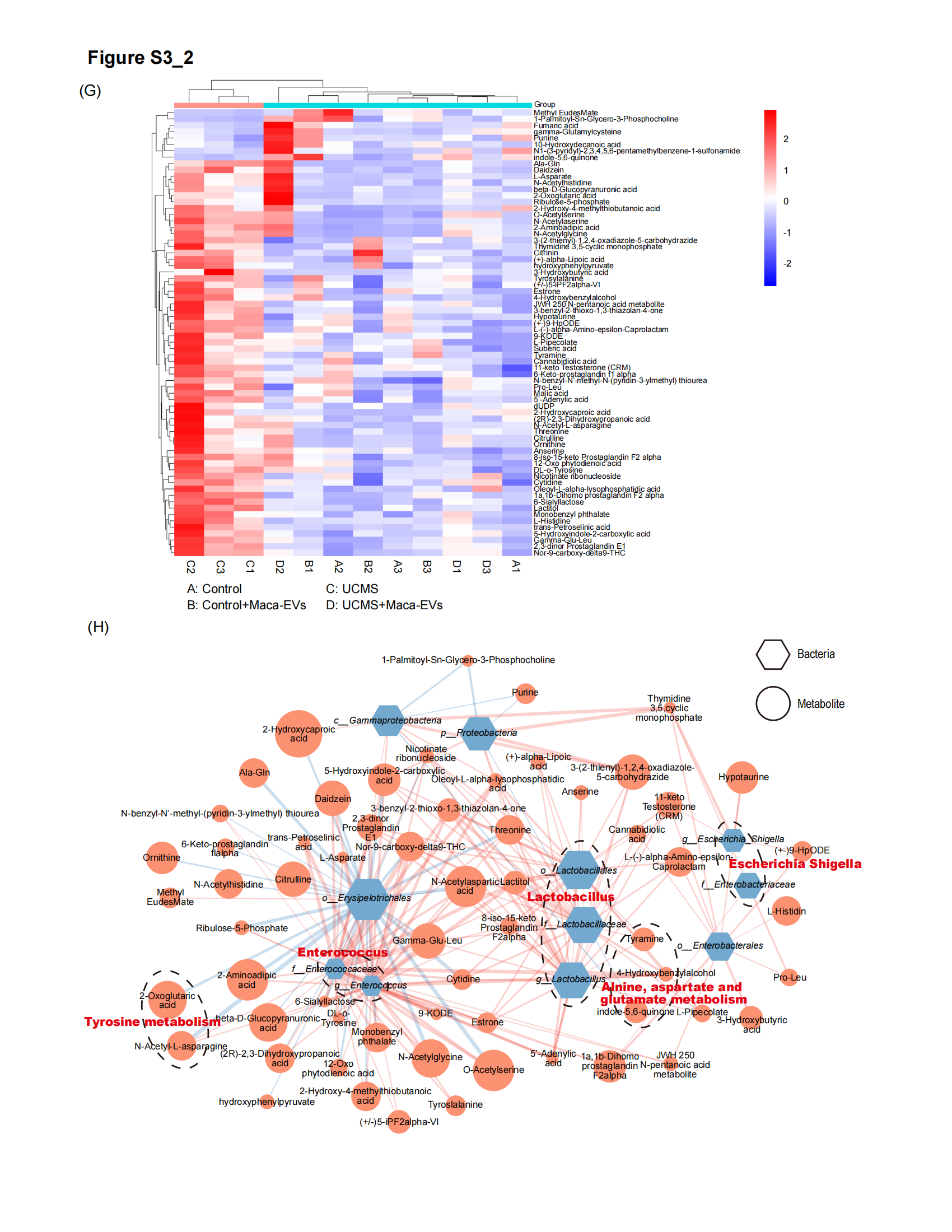


**Figure S4. Maca-EVs modulated the BDNF/GTP-Cdc42/ERK and TrkB/AKT signaling in the cortex of mice.** (A) Representative immunofluorescence images on the BDNF expressions in the cortex of control, Maca-EVs treated (200 μg/kg) or untreated UCMS mice. Scale bar: 50 μm. Western blot images and quantitative data on the expressions of BDNF (B), Cdc42 enzyme activity (C), p-ERK and ERK (C), Trkb (D), p-AKT and AKT (E) in the cortex of control, Maca-EVs untreated or treated (200 μg/kg) UCMS mice. (F) Representative immunofluorescence images on the NeuN cells in the cortex of control, Maca-EVs treated (200 μg/kg) or untreated UCMS mice. Scale bar: 50 μm. All data are presented as mean ± SEM (n=3 experiments for each group). Significance was evaluated by ordinary one-way analysis of variance (ANOVA) followed by the Turkey’s multiple comparisons test in (B, C, D, E). ns, not significant, *p* > 0.05, **p* < 0.05, ***p* < 0.01, ****p* < 0.001 between two indicated groups.


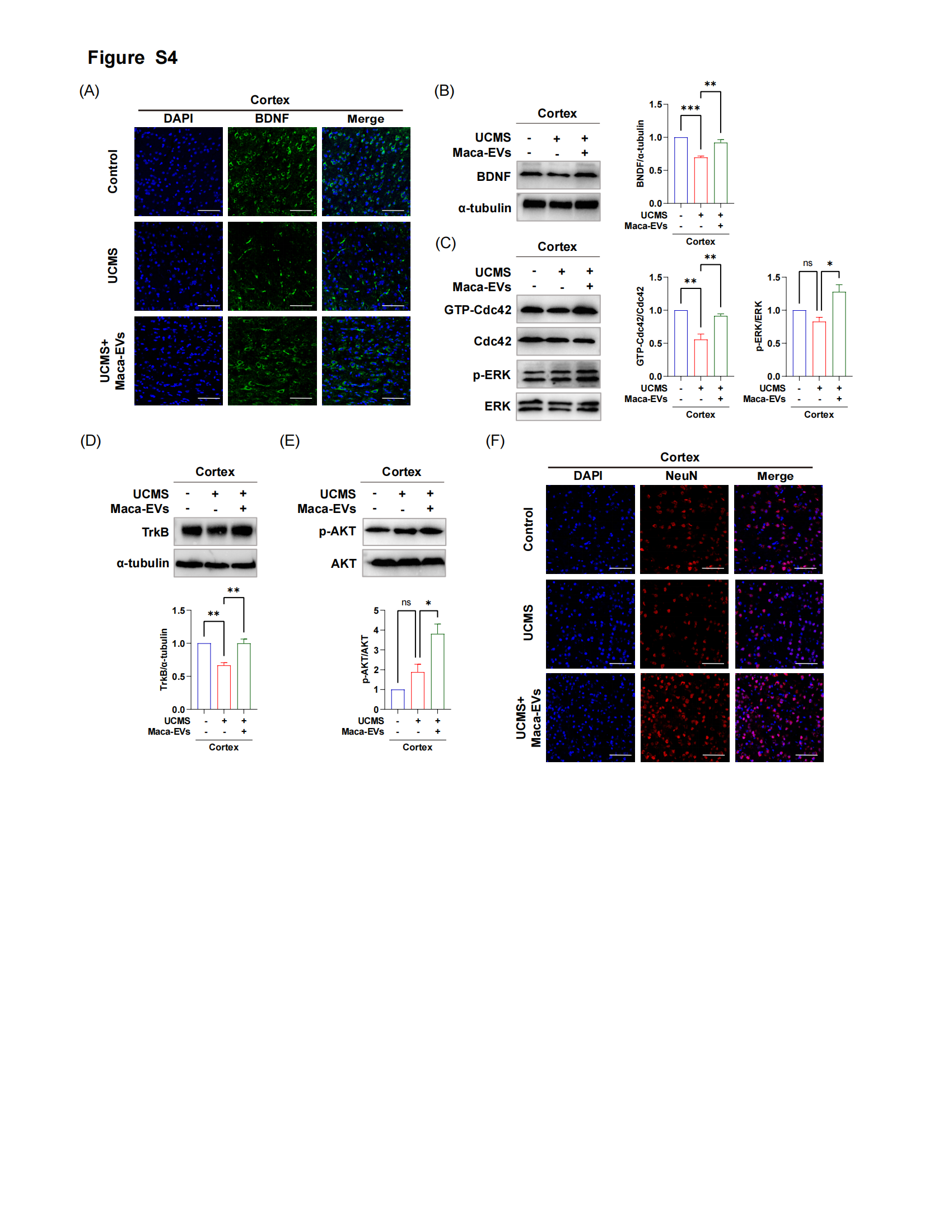

Supplement: Supplementary file 1 — Supporting information. [file IMT2-2-e116-s001.docx]
